# Cloning, Nucleotide Sequencing and Bioinformatics Study of NcSRS2 Gene, an Immunogen from Iranian Isolate of *Neospora caninum*

**Published:** 2013

**Authors:** M Soltani, A Sadrebazzaz, M Nassiri, M Tahmoorespoor

**Affiliations:** 1 Department of Biotechnology, Institute of Science and High Technology and Environmental Sciences, Graduate University of Advanced Technology, Kerman, Iran; 2 Razi Serum and Vaccine Research Institute, Mashhad, Iran; 3 Department of Animal Sciences, College of Agriculture, Ferdowsi University of Mashhad, Mashhad, Iran

**Keywords:** *Neospora caninum*, NcSRS2, Cloning, Sequencing, Iran

## Abstract

**Background:**

Neosporosis is caused by an obligate intracellular parasitic protozoa *Neospora caninum* which infect variety of hosts. NcSRS2 is an immuno-dominant antigen of *N. caninum* which is considered as one of the most promising targets for a recombinant or DNA vaccine against neosporosis. As no study has been carried out to identify the molecular structure of *N. caninum* in Iran, as first step, we prepared a scheme to identify this gene in this parasite in Iran.

**Methods:**

Tachyzoite total RNA was extracted and cDNA was synthesized and NcSRS2 gene was amplified using cDNA as template. Then the PCR product was cloned into pTZ57R/T vector and transformed into *E. coli* (DH5α strain). Finally, the recombinant plasmid was extracted from transformed *E. coli* and sequenced. Bioinformatics analysis also carried out.

**Results:**

The PCR product of NcSRS2 gene was sequenced and recorded in GenBank. The deduced amino acid sequence of NcSRS2 in current study was compared with other *N. caninum* NcSRS2 and showed some identities and differences.

**Conclusion:**

NcSRS2 gene of *N. caninum* successfully cloned in pTZ57R/T. Recombinant plasmid was confirmed by sequencing, colony PCR and enzymatic digestion. It is ready to express recombinant protein for further studies.

## Introduction

Bovine neosporosis is the most frequently diagnosed cause of bovine abortion worldwide (1). *Neospora caninum*, a persistent protozoan parasite capable of infecting almost any warm-blooded vertebrate, is a member of phylum apicomplexa and has a complex lifestyle involving two phases of growth: an intestinal phase in canine hosts, and an extra-intestinal phase in other mammals (2). It was originally identified in tissues of paralyzed dogs (3, 4). As revealed by molecular analyses, *N. caninum* is closely related to other coccidian parasite, *Toxoplasma gondii*, and therefore many of previously described *T. gondii* biological characteristics can be attributed to *N. caninum* so they would employ similar mechanisms for adhesion and invasion processes (2). Results of studies on *N. caninum* infection in Iran showed that this parasite could be considered as a cause of economic loss in dairy cattle (5). From several areas in Iran, *Neospora* infection has been reported in cattle (6–10), dogs (11–14) and camels (15, 16).

Current studies on *N. caninum* are mainly focused on the mechanisms and antigens involved in the tachyzoite adhesion, invasion and its proliferation and persistence in the host cell and using these antigens for immunological purposes (2). The surface proteins of the parasite are necessarily important to survival, since they start the interactions between the pathogen and the host cell surface molecules and the host immune response (17, 18).

The surface of *N. caninum* is coated with a family of glycosylphosphatidylinositol (GPI)-linked proteins (SRSs) (19). Attachment of the parasite to host cells and interface with the host immune response is mediated by SRS proteins to regulate the virulence of the parasite (19). One of surface protein molecules identified in tachyzoites of *N. caninum* is NcSRS2 which is shown to be most functionally involved in the adhesion and invasion process (20). It has been suggested that NcSRS2 provides some protection against experimental *N. caninum* infection (21, 22). Thus, this protein could be considered as a potential vaccine candidate against neosporosis in farm animals. Moreover, the immunogenicity of NcSRS2 has led to investigation of its using as a diagnostic reagent.

In the framework of the investigations on designing recombinant vaccines against neosporosis, this work focuses on the cloning and sequencing of NcSRS2 and bioinformatics based characterization of the important properties of its deduced protein. This work is first step in an attempt to design vaccine studies against neosporosis using NcSRS2 antigen that will be studied in the future.

## Materials and Methods

### Production of N. caninum tachyzoites

All cell culture reagents were purchased from Gibco-BRL (Zurich, Switzerland) and chemicals were from Sigma (St. Louis, MO, USA). Vero cells were routinely cultured in 25 cm^2^ tissue culture flasks in 5 ml of RPMI 1640 medium supplemented with 10% heat-inactivated FCS, 2 mM glutamine, 50 U of penicillin / mL and 50 ug of streptomycin / ml and incubated at 37°C with 5% CO_2_. A strain of *Neospora caninum* was kindly provided by Dr. Sadrebazzaz (Razi Vaccine and Serum Research Institute, Mashhad branch). This strain was isolated from an infected Holstein dairy herd in Mashhad. *N. caninum* cells which were maintained in BALB/c mice by serial intraperitoneal inoculation were used for the experiment. *N. caninum* tachyzoites were maintained by serial passages in Vero cells. Cultures were passaged at least once per week. When 80% of the Vero cells that had been infected with *N. caninum* tachyzoites shows cytopathic effect (typically 3-4 days p.i.), the monolayer cells were removed by scraping, twice washed with phosphate buffered saline (PBS) solution, and then centrifuged at 1000g for 10 min. Purified tachyzoites were checked for viability using trypan blue staining. Infected cells were trypsinized, washed twice in cold RPMI 1640 medium and the resulting pellet was resuspended in 2 ml cold RPMI 1640 medium. Cells were repeatedly passed through a 25G needle.

### RNA isolation and first strand cDNA synthesis

Total RNA was isolated from 2 × 10^6^ purified *N. caninum* tachyzoites using NucleoSpin^®^ RNA II kit (Machery-Nagel, Germany) according to the manufacturer's instructions. RNA concentration was measured with the NanoDrop ND1000 (Thermo Scientific, Delaware, US) system.

Single-stranded cDNA was synthesized from isolated total RNA using a cDNA synthesis kit (RevertAid™ First Strand cDNA Synthesis Kit, Fermentas, Germany) according to the standard protocol for first strand cDNA synthesis. Briefly, first strand cDNA synthesis reaction was performed in a 20 µl reaction mixture containing 100 ng of total RNA, 4 µl 5X reaction buffer, 2 µl 10 mM dNTP Mix, 12 µl nuclease-free water, 1 µl RiboLock™ RNase Inhibitor (20 u/µl), 1 µl RevertAid™ M-MuLV Reverse Transcriptase (200 u/µl) and 15 pmol of gene specific primers. Reaction mixtures were incubated for 5 minutes at 25°C followed by 60 minutes at 42°C and the reactions were terminated by heating at 70°C for 5 minutes.

### PCR amplification

To amplify NcSRS2 gene, one pair of gene-specific primers were designed using Primer Premiere software (Biosoft) based on published NcSRS2 gene sequence the GenBank. Primers were synthesized by Bioneer (South Korea) as follows:

NS21-F (5′-ACGAATTCATGGCGACGCATGCTT-3′) and NS21-R (5′-GCGTCGACTCAGTACGCAAAGATT-3′). PCR reactions were performed using total cDNA as template. Reaction was carried out in 25 µl volume containing approximately 100 ng of cDNA template, 50 mM Tris buffer (pH 8.3), 1.5 mM MgCl_2_, 0.2 mM of each of the 4 dNTPs, 0.5 U of *Pfu* DNA polymerase and 100 pM of each primers. Amplification reaction was performed using the following thermal profile: 95°C for 5 min, 35 amplification cycles (94°C for 50 sec., 61°C for 50 sec., and 72°C for 50 sec.), followed by a 72°C final extension for 10 min. Furthermore, false-negative results, caused by inhibitory compounds in the PCR reactions, were excluded by performing a simultaneous positive control reaction using the DNA extracted from tachyzoites of the NC-1 strain. The negative control consisted of dH_2_O without DNA. A positive and negative control was included in each reaction. Amplified PCR products were analyzed by electrophoresis of 5 µl of each sample on 1% (W/V) agarose gel at a constant voltage of 100 v for 40 minutes, stained with SYBR^®^ Safe DNA Gel Stain (Invitrogen, Paisley, UK). GeneRuler^TM^ 100 bp Plus DNA Ladder (Fermentas) was used to compare the DNA fragment sizes. Agarose gel illuminated under UV, and photographed with an UVidoc Gel Documentation System (UVitec, UK).

### Gel extraction of PCR products

The specific amplicons containing desired gene sequences were purified from the agarose gel by QIAquick Gel Extraction Kit (Qiagen, Germany) based on manufacturer's recommendations. This kit follows a simple bind-wash-elute procedure. Gel slices were dissolved in a buffer containing a pH indicator, allowing easy determination of the optimal pH for DNA binding, and then mixtures were applied to the QIAquick spin column. Nucleic acids adsorbed to the silica membrane in the conditions provided by the buffer. Impurities were washed away and pure DNA was eluted with a small volume of low-salt buffer provided.

### A tailing of PCR products

Since *Pfu* DNA polymerase possesses 3’ to 5’ exonuclease proofreading activity, the enzyme removes the 3′-A overhangs necessary for TA cloning, 3′-A overhangs must be added to fragments taking advantages of non-template activity of Taq DNA polymerase after PCR amplification since Taq polymerase preferentially adds an A to the 3’-ends in the presence of all four dNTPs. Briefly, a reaction was set up containing 25 µl purified PCR product, 5 µl 10 X Taq reaction buffer, 5 µl MgCl_2_, 5 µl dNTP (10mM stock), 1 µl Taq polymerase, 9 µl H_2_O. Then the mixture was incubated at 70 °C for 30 min. Finally, 3 µl of reaction mixture was run on a gel to quantify. This reaction product can directly used in ligation reaction without any need to clean up reaction.

### Ligation into pTZ57R/T vector

Tailed PCR products were ligated into pTZ57R/T Vector (Fermentas, Germany) based on TA cloning scheme according to the manufacturer's instructions. A 1:3 (vector to insert) molar ratio was used. Ligation reaction sat up in 30 µl volume containing 3 µl pTZ57R/T plasmid, 10 µl of A tailed PCR product, 1 µl T4 DNA ligase enzyme, 6 µl 5X buffer and 10 µl nuclease free distilled water. After gentle mix and a brief centrifuge, the ligation reaction mixture was incubated overnight at 10 °C. The resulting plasmid was designated as pTZ-NcSRS2. Recombinant vectors were stored at -20°C until transformation.

### Transformation, Screening and Colony PCR

Preparation of competent cells from *E. coli* strain DH5-α was performed by calcium chloride method (23). Advantages of chemical preparation of competent cells include simple procedure; no special equipment required and gives good transformation efficiencies. In general, it is the best method to use when the transformation efficiencies is not the problem. To transform competent cells, 10 µl of ligation reaction product was added to 150 µl of competent cells and placed on ice for 40 minutes after vortex and spin. Then the mixture was incubated at 42 °C for 90 s and immediately was placed on ice for 5 minutes. Then 1 ml of LB antibiotic free medium was added to the transformed cells and allowed to recover by incubation at 37 °C for 2 hours with shaking. Cells harboring pTZ-NcSRS2 plasmid were plated and grown overnight at 37 °C on LB agar plates (10 g NaCl, 5 g yeast extract, 10 g bacto tryptone) with ampicillin (100 µg/ml), X-Gal (Fermentas) and IPTG (Fermentas) for blue-white screening. After overnight incubation, plates were placed at 4 °C for 2 hours and cells from white colonies were harvested and cultured on antibiotic containing LB agar plates. After 16 hours incubation at 37 °C, cells harboring the recombinant plasmids grew up. Recombination confirmed by colony PCR with NcSRS2 gene specific primers. This technique was used to determine insert size in the vector. Briefly, a colony was picked with toothpick and swirl into 50 µl of ddH2O in a 1.5 ml microfuge tube. Then heat the tubes at 95 °C for 10 minutes. Tubes were centrifuged for 5 minutes at top speed in microfuge and 40 µl of supernatant was transferred to new 0.5 ml microfuge tubes and 2 µl of it was used as template in PCR reaction. All other PCR reaction conditions were as before.

### Plasmid Purification

Cells harboring the recombinant plasmid were cultured in antibiotic containing LB medium for 16 hours at 37 °C in a shaker incubator. GeneJET Plasmid Miniprep Kit (Fermentas) was used to purify recombinant plasmids from *E. coli* DH5α following the manufacturer's instructions. Briefly, 4 ml bacterial culture was harvested and lysed. The lysate was then cleared by centrifugation and applied on the silica column to selectively bind DNA molecules. The adsorbed DNA was washed to remove contaminants, and the pure plasmid DNA was eluted in a small volume of elution buffer. Plasmid DNA concentrations were determined by absorbance at 260 nm using NanoDrop ND1000 (Thermo Scientific, Delaware, US) system. The integrity of the DNA plasmids was checked by agarose gel electrophoresis. Also resultant pTZ-NcSRS2 recombinant plasmids were compared with native plasmid (pTZ57R/T) by electrophoresis of 3 µl of extracted plasmid on a 1% agarose gel.

### Enzyme Digestion of pTZ-NcSRS2

With regard to presence of *EcoR*I and *Sal*I restriction sites on recombinant plasmids extracted from white colonies, the recombinant plasmids were characterized for the presence and size of inserts by double digestion. Each 20 µl digestion reaction contained 10 µl of recombinant plasmid, 10 units of each restriction enzyme, 2 µl of 10X buffer (buffer O) and 6 µl of dH_2_O. Digestion was performed by incubation at 37 °C for 2 hours. Digestion products were analyzed by electrophoresis on 1% agarose gel containing SYBR^®^ Safe DNA Gel Stain (Invitrogen, Paisley, UK).

### Sequencing of NcSRS2 gene

The nucleotide sequence of the inserts (NcSRS2) in the recombinant plasmid (pTZ-NcSRS2) was verified by sequencing in the forward and reverse directions using primer walking approach (Eurofins MWG Operon, Germany). M13 uni (-21) forward primer (5′-TGTAAAACGACGGCCAGT-3′) and M13 rev (-29) reverse primer (5′-CAGGAAACAGCTATGACC-3′) were used for sequencing. DNA Baser v3 (Heracle BioSoft, Romania) was used for sequencing data assembly to produce a consensus sequence for each DNA sample used.

### Blast search and bioinformatics study

The nucleotide sequence of NcSRS2 was submitted to the BLAST search (megablast algorithm) at NCBI server (http://www.ncbi.nlm.nih.gov/blast/) to compare with sequences presented in the GenBank. For detailed analysis, all closely related sequences and deduced amino acid sequences between published sequences were aligned by ClustalW2 multiple sequence alignment program (http://www.ebi.ac.uk/Tools/clustalw2/) (24). ClustalW2 is a general tool used for multiple alignments of DNA sequences. The sequences were analyzed for signal peptides using SignalP 4.0 (http://www.cbs.dtu.dk/services/SignalP/) (25), protein domains using Prosite (http://prosite.expasy.org/) (26) and potential transmembrane regions were checked with the ProtScale tool on the Expasy server (http://expasy.org/tools/protscale.html).

Hydrophobicity plot of NcSRS2 protein was also drawn which characterizes its hydrophobic and hydrophilic characteristics that may be useful in predicting membrane-spanning domains, potential antigenic sites and regions that are likely exposed on the protein surface (27, 28).

Phylogenetic and molecular evolutionary analyses were conducted using CLC main workbench software (CLC bio) by bootstrap test with 1000 replications was applied to estimate the confidence of branching patterns of the UPGMA tree. Also, a pairwise comparison was done to clarify the pairwise distances and percent identities.

## Results

### Production of N. caninum tachyzoites

**Fig. 1:**
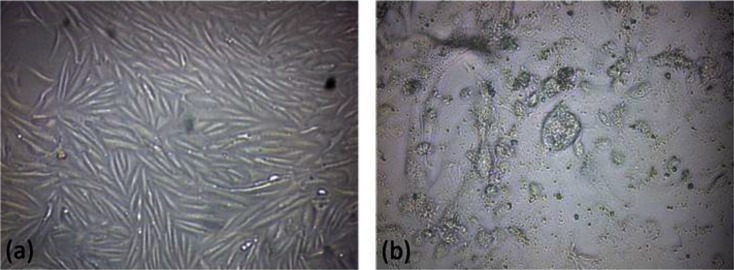
(a) Confluent Vero cells on day 3. (b) *N. caninum* tachyzoite infected Vero cells

Vero cells became confluent on day 3 and then were infected with *Neospora caninum* tachyzoites. Tachyzoites grew well in Vero monolayers (Fig. 1). *N. caninum* tachyzoites were maintained in and purified from, Vero cell monolayers and were immediately used for RNA extraction.

### RNA isolation and first strand cDNA synthesis

Extracted RNA samples had very good quality and integrity based on NanoDrop analysis results. The OD 260/280 ratio for purified RNA was between 1.80–1.95, indicating that preparations were free of any major protein contamination. NanoDrop results showed that first strand cDNA synthesis reaction was successful.

### PCR amplification

**Fig. 2:**
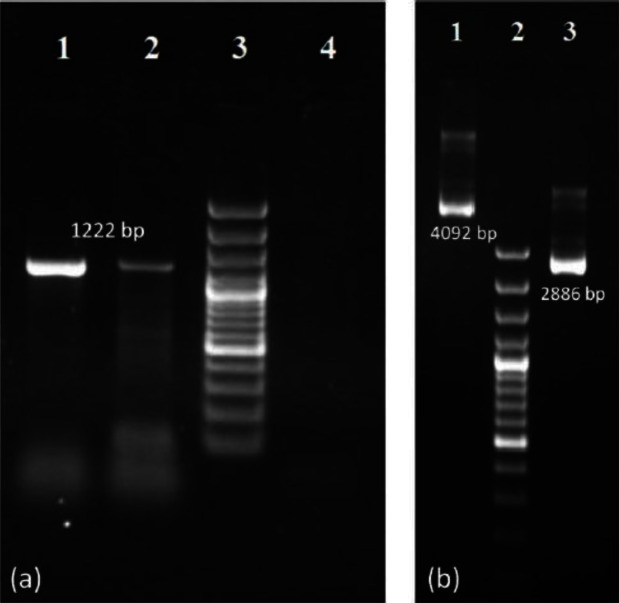
(a) Amplification of synthesized cDNA with the specific primer pairs for NcSRS2 (lane 1: positive control, lane 2: NcSRS2 cDNA, lane 3: M100 bp plus size marker, lane 4: negative control). (b) Comparison of plasmids on 1% agarose gel (lane 1: pTZ-NcSRS2, lane 2: M100 bp plus size marker, lane 3: native pTZ57R/T)

As PCR results shows in Fig. 2-a, synthesized cDNA were successfully amplified by PCR reaction. The presence of amplicon is characteristic for the presence of the *N. caninum* DNA. NcSRS2 PCR reaction yielded a specific 1222 bp long product. The intensity and size of resulted band was identical with *N. caninum* (NC-1) positive control that confirmed the accuracy of performed reactions. Furthermore, no visible bands can be seen in negative control lanes. PCR product was used for ligation into pTZ57R/T vector after A-tailing process.

### Comparison of native and recombinant plasmids

A-tailed PCR products were ligated into pTZ57R/T vector using TA cloning scheme. Resultant recombinant plasmid (pTZ-NcSRS2) was compared with native pTZ57R/T by electrophoresis on 1% agarose gel (Fig. 2-b). As expected, pTZ-NcSRS2 (4092 bp) was longer than native pTZ57R/T (2886 bp). Different bands revealed in each plasmid lanes can be attributed to different forms of extracted plasmid DNA (linear, open circular and supercoil).

### Colony PCR and Enzyme digestion

**Fig. 3:**
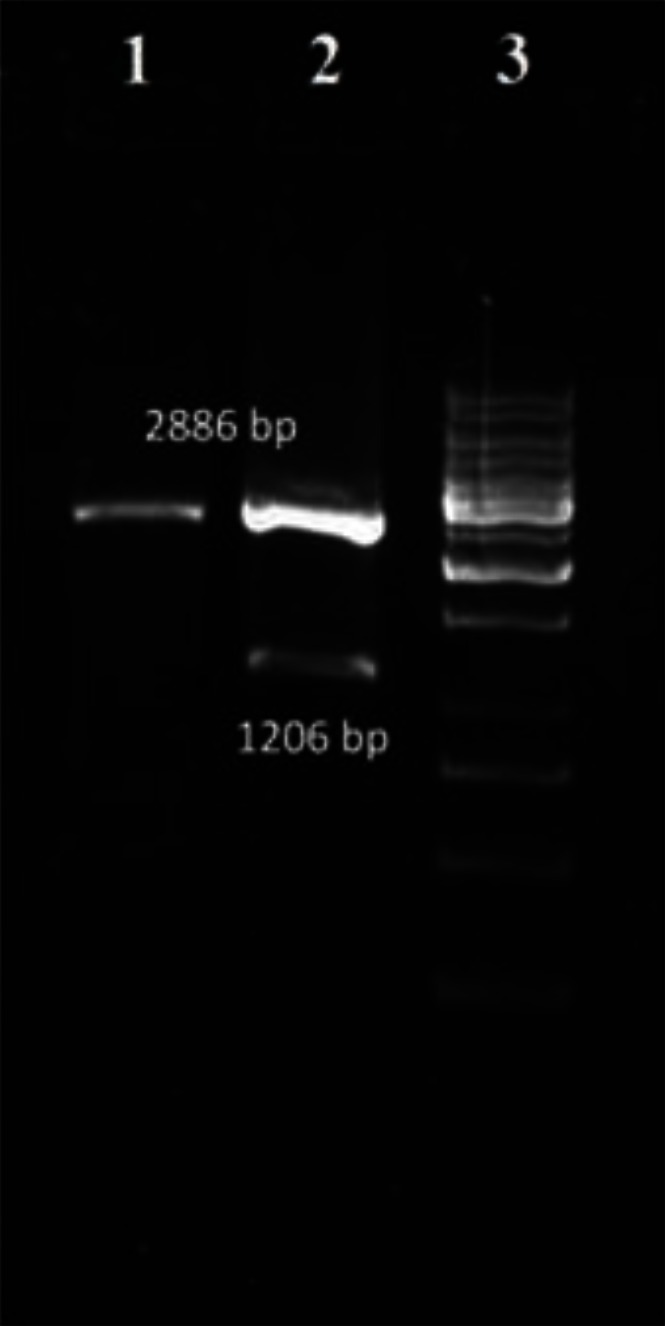
Agarose Gel electrophoresis of restriction enzyme digested recombinant plasmid pTZ-NcSRS2. lane 1: native pTZ57R/T, lane 2: EcoRI/SalI digested pTZ-NcSRS2, lane 3: 1 kb DNA size marker

Colony PCR was used to confirm the recombination. Colony PCR was performed using NcSRS2 gene specific primers. Selected white colonies generated strong bands after PCR that showed recombination process was done as expected. To further confirm presence and size of inserts in pTZ-NcSRS2, recombinant plasmid was simultaneously digested with two enzymes (EcoRI/ SalI). After electrophoresis of digestion reaction on 1% agarose gel, 2 bands were detected in each lane that can be attributed to pTZ57R/T band (2886 bp) and insert band (1206 bp for NcSRS2). As shown in Fig. 3, inserts of expected length were detected.

### Sequencing of NcSRS2

NcSRS2 gene generated by PCR was successfully cloned and sequenced. Sequence data reported in this paper is available in the GenBank database under the accession numbers JQ410454.

**Fig. 4:**
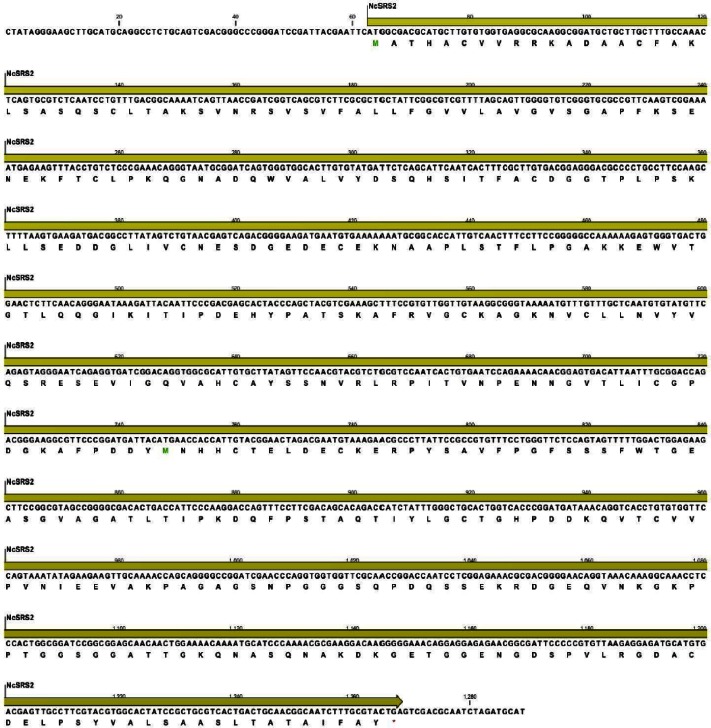
Nucleotide and translated sequence of NcSRS2

Protein encoded by NcSRS2 genes had length of 401 amino acids and molecular mass of 42 kDa, based on the in silico estimates using CLC main workbench software package (CLC bio), and was similar to the NcSRS2 protein sequences obtained from the *Neospora caninum* homepage on GeneDB (http://www.genedb.org/Homepage/Ncaninum) (Fig. 4). The proteins correspond to gene models as follows: NcSRS2 (NCLIV_033250).

### Bioinformatics study

Sequence analysis of NcSRS2 gene cloned in pTZ57R/T shows that this sequence has between 98-100% identities with other recorded NcSRS2 genes in GenBank. A 91% identity with *N. hugeshi* also could be observed. Similarity of this gene with *T. gondii* SRS genes varied between 31-42%.

Various physico-chemical properties of studied proteins were computed using ProtParam program. ProtParam analysis results are shown in Table 1.

**Table 1:** Physico-chemical properties of NcSRS2 protein derived from ProtParam

Number of amino acids	401
Molecular weight	42009.9
Theoretical pI	5.28
Negatively charged residues (Asp + Glu)	45
Positively charged residues (Arg + Lys)	35
Formula	C 1824 H 2865 N 507 O 596 S 18
Total number of atoms	5810
Extinction coefficients	30910 (29910)
Estimated half-life	30 hours (mammalian reticulocytes, in vitro)
	>20 hours (yeast, in vivo)
	>10 hours (Escherichia coli, in vivo)
Instability index	49.24
Aliphatic index	69.35
GRAVY	-0.294

UV spectrophotometry is a useful tool for determining protein concentration in a solution. In order to take advantage of this method one needs an accurate measure of the protein of interest's extinction coefficient (molar absorption coefficient). The extinction coefficient indicates how much light a protein absorbs at a ertain wavelength. This estimation is useful for following a protein with a spectrophotometer when purifying it. Two values are produced by ProtParam, both for proteins measured in water at 280 nm. The irst one shows the computed value based on the assumption that all cysteine residues appear as half cystines (i.e. all pairs of Cys residues form cystines), and the second one assuming that no cysteine appears as half cystine (i.e. assuming all Cys residues are reduced). This measure is estimated using the method of Pace et al., which calculates the sum of (NumberAA x Extinction CoefficientAA) for three amino acids that absorb at 280 nm: tyrosine, tryptophan, and the dimeric amino acid cystine (two cysteine [Cys] residues covalently joined through a disulfide bond. The absorbance of the protein at 280 nm (A280, or OD280) is calculated by dividing the extinction coefficient by the molecular weight of the protein.

The half-life is a prediction of the time it takes for half of the amount of protein in a cell to disappear after its synthesis in the cell. ProtParam relies on the "N-end rule", which relates the half-life of a protein to the identity of its N-terminal residue; the prediction is given for 3 model organisms (human, yeast and E. coli). The N-end rule states that the in vivo half-life of a protein is a function of the nature of its amino-terminal residue (29). Because the N terminus amino acid of NcSRS2 protein is Methionine, so based on N-end rule, it will be stable more than 10 hours in *E. coli* cells.

The instability index provides an estimate of the stability of protein of interest in a test tube. Values greater than 40 indicate that the protein may be unstable in vitro. This index is calculated using the method of Guruprasad et al. (30), which assigns a weighted instability value to each dipeptide in the protein. These values were derived from an analysis that found a significant difference in the occurrence of certain dipeptides between stable and unstable proteins. Based on ProtParam analysis results, NcSRS2 is classified as an unstable protein (Instability index: 49.24).

**Fig. 5:**
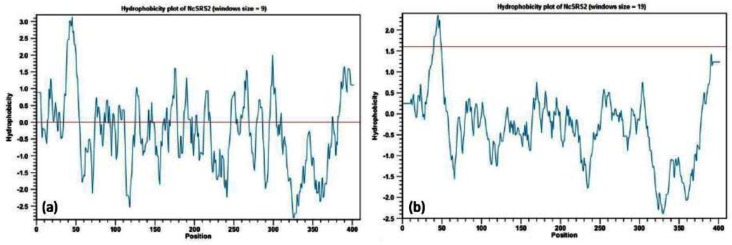
Hydrophobicity plots of the NcSRS2 (a) windows size = 9, (b) windows size = 19.

**Fig. 6:**
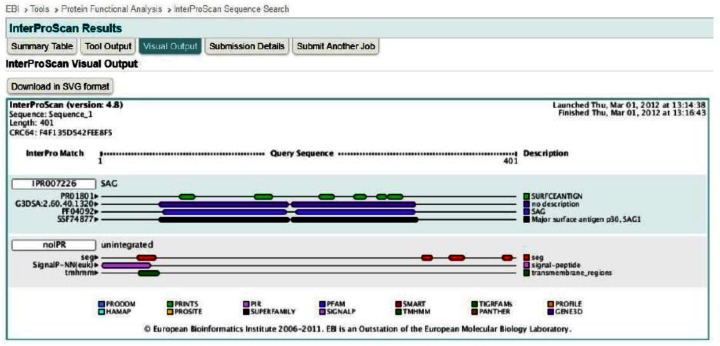
InterProScan visual output for NcSRS2

The protein aliphatic index is defined as the relative volume occupied by aliphatic side chains (alanine, valine, isoleucine, and leucine). It may be regarded as a positive factor for the increase of thermostability of globular proteins. Aliphatic index is calculated using the method of Ikai as the sum of (Molar%AA x Volume AA) for alanine, leucine, isoleucine and valine (where Volume AA is the relative value compared to alanine). Aliphatic index analysis results was consistent with previously described results from instability index while aliphatic index is defined as a measure of thermostability, lower value of instability index for NcSRS2 can be contributed to aliphatic index. In other words, higher aliphatic index is related to lower instability index and higher stability. A GRAVY (Grand Average of hydropathicity) score can be calculated as the sum of the hydropathy values for all the amino acids in a protein sequence divided by the number of residues in the sequence. In essence, a GRAVY score is the relative value for the hydrophobic residues of the protein. Although no positional or interaction effects for adjacent residues are taken into consideration by the GRAVY score, it still provides some indication of the physical state of the protein. This index indicates the solubility of the proteins: positive GRAVY protein is hydrophobic while negative GRAVY protein is hydrophilic. As derived from ProtParam analysis, NcSRS2 gained a negative GRAVY score so it can be inferred that NcSRS2 is a hydrophilic protein. Integral membrane proteins typically have higher GRAVY scores than do globular proteins. Though this score is another helpful piece of information, it cannot reliably predict the structure without the help of hydropathy plots. There are some methods for evaluation of the degree of interaction of polar solvents such as water with specific amino acids. In these methods a hydrophobicity plot is created that is a quantitative analysis of the degree of hydrophobicity or hydrophilicity of amino acids in a protein (Kyte-Doolittle scale indicates hydrophobic amino acids, while the Hopp-Woods scale measures hydrophilic residues). This measure is implicated to identify possible structure or domains of a protein. Plot shape analysis prepares information about partial structure of the protein of interest. For example, extension of about 20 amino acids with positive shows that these amino acids may be part of alpha-helix spanning across a lipid bilayer, which is composed of hydrophobic fatty acids. On the other hand, stretch of amino acids with negative hydrophobicity indicates that these residues are in contact with solvent or water, and that they are probably resided on the outer surface of the protein. To elucidate antigenic properties of NcSRS2, the hydrophobicity plot of the deduced sequence was reproduced (Fig. 5). Two plots were drawn for NcSRS2; one of them was plotted with windows size of 9 for seeking surface regions and second one was plotted with windows size of 19 to look for transmembrane regions. As shown in figure 6-a, possible surface regions can be identified as strong negative peaks. In figure 6-b, transmembrane regions are identified by peaks with scores greater than 1.6.

InterProScan results are also summarized in Fig. 6 and Table 2.

**Table 2:** NcSRS2 confidently predicted domains, repeats, motifs and features

Name	Begin	End	E-value
Transmembrane	35	54	-
Pfam:SAG	59	180	2.00e-13
Pfam:SAG	187	303	1.20e-23
Low complexity	309	320	
Low complexity	336	351	
Low complexity	387	397	

Surface antigen genes are spread throughout the *N. caninum* genome with little polymorphism at each locus. They can be divided into two groups termed SAG1 or SAG2. SAG1 surface protein family is crucial for virulence. SAG1 is composed of two disulphide linked SRS domains. These have 6 cysteines that form 1-6, 2-5 and 3-4 pairings.

**Fig. 7:**
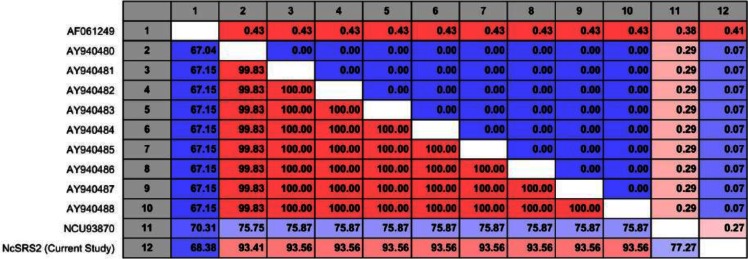
Upper diagonal: Calculated pairwise Jukes-Cantor distance, Lower diagonal: Calculated pairwise percent identities

Jukes-Cantor distance between each pairs of sequences was calculated (Fig. 7 – upper diagonal). This number is given as the Jukes-Cantor correction of the proportion between identical and overlapping alignment positions between the two sequences. Also percent identity calculated as the percentage of identical residues in alignment positions to overlapping alignment positions between each pair of sequences (Fig. 7 – lower diagonal).

**Fig. 8:**
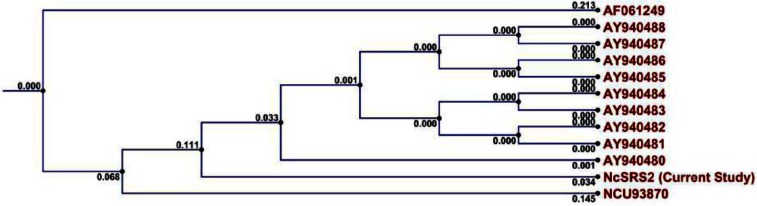
Phylogeny of NcSRS2 sequence of the current study. The tree was constructed using the UPGMA method. Numbers along branches represent length values.

To determine the phylogenetic position of the NcSRS2 in the current study, the 1289 bp length of the sequence was used for comparative sequence analysis against known NcSRS2 sequences. The NcSRS2 sequence of the current study showed a high relationship to each of known sequences of the NcSRS2 (Fig. 8).

## Discussion

*Neospora caninum* is an obligatory intracellular parasite which has complicated life cycle and almost infects all nucleated cells (2). It causes dangerous manifestation in fetus which the most dangerous effect of congenital neosporosis is abortion (2, 6). The congenital infection has different symptoms based on the intensity and variety of contamination in the organs. Severity of the disease is related to stage of the pregnancy period which the infection occurs (5). In this study, the NcSRS2 gene of *N. caninum* tachyzoites surface antigen was cloned for studying its immunogenic potentials in future. Hemphill and Gottstein (31) identified major surface protein on *N. caninum* tachyzoites. Using postembedding immunogold labeling with anti-Nc-p43 antibodies, Hemphill (20) demonstrated that Nc-p43 is localized not only on the parasite cell surface but also within dense granules and rhoptries. Many of researchers have used this gene as a candidate vaccine component in different expression schemes like vaccinia virus (32–36), herpes virus (37), baculovirus (38) and DNA vaccine (22, 39) and conclude that this gene can be considered as a potent immunogen in vaccine studies against neosporosis.

Many of researchers have used NcSRS2 as a vaccine candidate in various expression systems like vaccinia virus, Herpes virus, Baculovirus, and DNA vaccine and have shown that this antigen can be used as a suitable target for vaccine design against neosporosis. A 1222 bp fragment corresponding to NcSRS2 gene was successfully cloned and sequenced and bioinformatics analysis on its deduced protein sequence was performed. Detection of virulence factors and their corresponding genes and antigens is the first step in vaccine designing against a pathogenic organism. Results of the current study, as the first molecular study on Iranian isolate of *N. caninum* revealed that there is no obvious difference between this isolate and other recorded *Neospora* isolates. These features are in agreement with data demonstrating that NcSRS2 is a GPI-anchored membrane protein (40). Prosite analysis showed a cell attachment sequence near the end of NcSRS2 sequence. The sequence Arg-Gly-Asp (RGD) is essential for interaction with cell surface receptor (41, 42) and so has a key role in *N. caninum* tachyzoite cell adhesion process. Phylogenetic analysis showed that studied isolate is closely related to worldwide *Neospora* isolates, so no specific pattern for distribution of the parasite can be envisaged.

## Conclusion

As revealed using bioinformatics analyses, NcSRS2 protein has suitable thermostability so it can be used as on farm vaccine formulations. By nucleotide description of NcSRS2, the surface immunogenic antigen of Iranian isolate of *N. caninum*, the way to design recombinant and DNA vaccines against neosporosis has been started. Cloning and expression of this gene in various hosts is the next step to prepare an effective vaccine formula against neosporosis.
